# Resveratrol-enriched rice identical to original Dongjin rice variety with respect to major agronomic traits in different cultivation years and regions

**DOI:** 10.1080/21645698.2021.1979368

**Published:** 2021-12-08

**Authors:** Vipada Kantayos, Woon-Chul Shin, Jin-Suk Kim, Seung-Ho Jeon, Eui-Shik Rha, So-Hyeon Baek

**Affiliations:** a Department of Agricultural Life Science, Sunchon National University, Suncheon, Republic of Korea; b Bioenergy Crop Research Institute, NICS, RDA, Muan, Republic of Korea

**Keywords:** agronomic trait, high-performance liquid chromatography, resveratrol, rice, Southern blot

## Abstract

Resveratrol is synthesized by the catalysis of resveratrol synthases (RS) in a limited number of higher plants. Resveratrol shows potential health-promoting properties, including as an antioxidant and in preventing cardiovascular diseases. Recently, resveratrol-enriched rice has been produced as a novel source of resveratrol. This study aimed to investigate the major agronomic characteristics of resveratrol-enriched rice, Iksan526 (I526) and compared them with those of a nontransgenic and commercial rice variety, Dongjin (DJ). Transgene (RS) integration was confirmed using Southern blot analysis, and homologous recombination was achieved after digestion with the SacI restriction enzyme. The phenotypic traits of I526 grown in Iksan were similar to those grown in Milyang but not similar to those grown in Suwon. In Suwon, I526 had slightly earlier heading dates [i.e., number of days from sowing to heading) and shorter culm lengths. When I526 was treated with 0.4% Basta in the seedling stage, no significant difference was observed among all the agronomic traits compared with nontreated I526; particularly, the culm length, panicle length, number of panicles per hill, 1,000 grain weight of brown rice, and brown rice yield of the Basta-treated rice were similar to those of the nontreated I526, regardless of their cultivation region. The resveratrol content of I526 grown in Suwon and Milyang was increased by 18% and 37%, respectively, than that of I526 grown in the Iksan area. Therefore, DJ and I526 are not significantly different in terms of major agronomic traits depending on variety/year and variety/cultivation region. The results indicated that I526 has the potential to become a commercialized variety in the near future.

## Introduction

Owing to the successful experiments of,^1^aimed at introducing the *Staphylococcus* gene to colon *Bacillus*, recombinant DNA technology can currently be used in microorganisms, animals, and plants. Since the commercialization of genetically modified (GM) herbicide-resistant beans in 1996, 526 types of events for 32 crops have been approved in 44 countries as of 2018. The region or area for the cultivation of GM crops across the globe has increased from 1.7 million ha in 1996 to approximately 191.7 million ha in 2018, which is equivalent to an increase of approximately 113 times. This region accounts for approximately 12.8% of the total 1.5 billion ha cultivation region across the globe. The aggregated cultivation region of GM crops with respect to countries is as follows: 71.5 million ha in the United States of America (US), 40.3 million ha in Brazil, 24.4 million ha in Argentina, and 1.10 million ha in India (ISAAA, 2018). Moreover, eight countries have a cultivation region of <50,000 ha. According to International Service for the Acquisition of Agri-biotech Applications, 18 million farmers in 26 countries cultivate GM crops and 16.5 million farmers (approximately 90%) have small-scale farms in developing countries. Recently, China and India are beginning to ease restrictions on the cultivation and import of GM crops to address their food shortage issues. In addition, five countries in the European Union, including Germany, have also initiated GM crop cultivation. As such, the governments of these countries have recently begun to allow the cultivation of GM crops despite opposition from consumers (ISAAA, 2018).

GM rice, the first crop approved for commercialization, is a herbicide resistant rice variety approved by the US government in 1999,^2^ and recently, vitamin A-enriched rice (golden rice) has been approved for commercial production in the Philippines. Moreover, research teams in Germany and India have developed GM rice showing a 22% increase in flavonoid content through the overexpression of anthocyanin synthase.^3^ In 2009, the Chinese Academy of Agricultural Sciences developed two kinds of rice, insect-resistant (Cry1Ac) Huahui-1 Bt rice and Shanyou-63 Bt rice; they also developed a marker-free event through crossbreeding after co-transforming a target gene and a selectable marker.^4^ In addition, in Switzerland, a new variety of rice containing six times more iron than existing varieties was developed by introducing two new genes.^5^

Resveratrol is a phytoalexin substance with defensive properties that is produced in plants after exposure to strong ultraviolet (UV) rays or gray mold.^6,7^ Resveratrol shows strong antioxidant activity and is being used in the treatment of diseases such as colon cancer, oral herpes, and ulcers. Moreover, its pharmacological efficacy in terms of health-promoting properties, such as the anticancer, antihyperlipidemic, and anti-inflammatory properties, has been reportedly observed to be effective for conditions including seizures and cardiovascular diseases.^8–11^ Recently, Iksan526 (I526), a resveratrol-enriched rice variety, was developed for the first time using the resveratrol synthase (RS) gene, I526 originates from Dongjin (DJ) rice, which is the most wildly cultivated rice variety in Korea. The RS gene used in the development of I526 is derived from Palkwang, a Korean peanut variety. Animal studies using mice models indicated that I526 is efficient in preventing metabolic syndromes and is effective in the treatment of conditions such as obesity, hyperlipidemia, and high cholesterol. ^12,13^

Genetic engineering is a tool for developing plants that produce specific metabolites as a novel source in functional food and in the pharmaceutical industry. In the past, GM rice was developed for improving the nutritional value of rice. Although GM rice is not approved for consumption in some countries, there is no evidence that GM rice consumption is harmful to humans.^14^ To cultivate GM rice for commercial purposes, productivity and the surrounding environment should be considered and evaluated before being approved for commercial use. Thus, it is important to assess the conditions of the cultivation area and agronomic traits of transgenic crop by comparison with its wild-type. Transgenic crop adaptation and transgene expression stability should also be verified. A potential crop base is generally evaluated for its agronomic traits to select crop types that can grow for a long term in an agricultural system. Environmental interactions that affect the genotype, which are assessed in field experiments in different locations and years, are also important in crop improvement programs.^15^

This study aimed to compare the major agronomic traits between I526 and its non-transgenic parent, DJ, in terms of cultivation year and region. This research was conducted to obtain data that can be used for the commercialization of resveratrol-enriched rice as a new pharmaceutical material.

## Materials and Methods

### Southern Blotting

The genetic stability of RS gene in I526 was verified using Southern blotting following the method described by.^16^ Genomic DNA (10 μg) was treated with SacI restriction enzyme. The concentrated DNA was then separated by electrophoresis on a 0.8% agarose gel at 25 V for 16 h. Subsequently, the DNA bands were transferred to Hybond N+ nylon membrane (Amersham, UK). Using Alkphos Direct Labeling kit (Amersham, UK), DNA probes were hybridized to the RS cDNA template to visualize the position of the transgene on the hybridized membrane. The hybridized membrane was developed by exposure to X-rays for 3 h, following which the results were confirmed.

### Investigation of the Agricultural Traits of I526 by Cultivation Region

I526 and DJ rice were cultivated in GM organism-confined fields in the Suwon, Iksan, and Milyang regions of the National Institute of Crop Science from 2013 to 2015, with a planting distance of 30 × 15 cm. In addition, N–P–K at 9–4.5-5.7 kg/10a was used as fertilizer; all other cultivation conditions followed here were in compliance with the standard rice cultural practices of the Rural Development Administration. The investigation of major agricultural traits, including growth analysis (culm length, panicle length, number of panicles/hill, and number of spikelets/panicles) and yield response (ratio of ripened grain, 1,000 grain weight of brown rice, brown/rough rice ratio, rough rice yield, and brown rice yield), was conducted following the standards of study, investigation, and analysis of Agricultural Science Technology of Rural Development Administration.^17^

Transgenic rice was screened for the transformation of the RS gene by applying 0.4% glufosinate ammonium (Basta^TM^, Bayer AG, Germany) at 2 weeks after planting. The major agronomic traits and heading dates (i.e., number of days from sowing to heading) of transgenic DJ526 were observed by comparing the nontreated (I526) and glufosinate ammonium-treated (I526B) I526 rice. The weather information service (http://weather.rda.go.kr/analysis/region.jsp) of Rural Development Administration was used to investigate the heading dates in the three regions.

### Comparison of Resveratrol Content of I526 by Cultivation Year and Region

The seeds were harvested from genetically modified organism-confined field trials in three locations, Suwon, Iksan, and Milyang. After dehusking seeds with mill grinder (Retsch MM301, Germany), fine powdered brown rice (300 mg) was used to examine resveratrol content; 80% methanol (90 µL) was added to the fine powder and the mixture was sonicated (UIL ultrasonic, Korea) at room temperature for 30 min. The extracts were centrifuged at 10,000 × *g* for 5 min at 4°C; the supernatant was filtered through a 0.2-µm nylon membrane filter; and 1 µL of the extract was analyzed for trans-resveratrol content using ultra-performance liquid chromatography (UPLC, ACQUITYTM Ultra Performance LC, Waters, USA) equipped with stationary ACQUITYTM UPLC BEH C18 column (2.1 × 100 mm ID, 1.7 µm, Waters, USA) and a UV detector (TUV detector, Waters, USA). Acetonitrile (A; in 0.1% formic acid) and 0.1% formic acid (B) in water were maintained for 10 min at a ratio 10:90 (A:B), for 12 min with the ratio changed to 15:85 and changed to 25:75 for 0.4 min. Subsequently, the ratio was maintained at 90:10 for 0.6 min and rebalance the column at 10:90 for 1 min. The UV wavelength of the detector was set to 308 mm.

### Statistical Analysis

Data were analyzed using ANOVA (PROC ANOVA procedure of SAS analytical software, V.9.3, Cary, North Carolina, USA), and the mean of agronomic traits and yield trait in the three regions during the 3 y of investigation were compared using the least significant difference test (LSD) at 5% and 1% probability levels.

## Results

### Genetic Stability of the RS Gene

**Figure 1.**
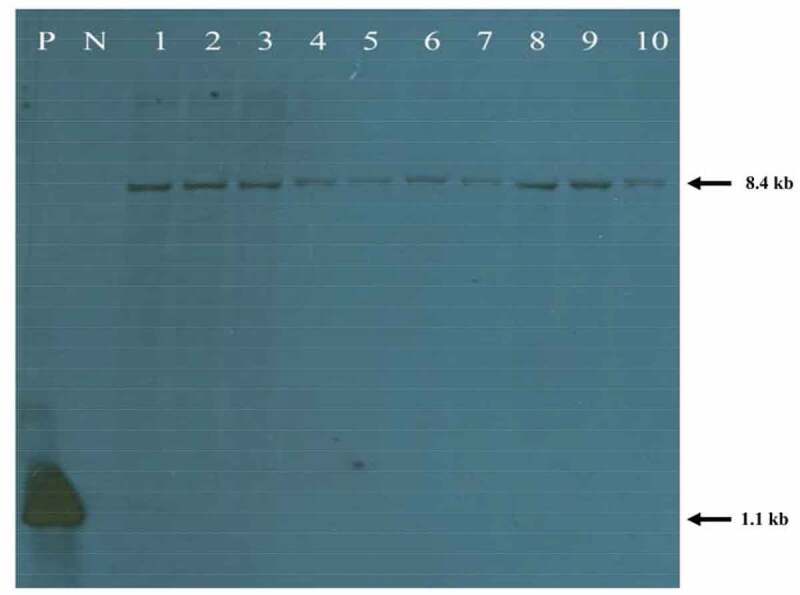
Southern blotting of the RS transgene in dongjin and Iksan526 lines

In total, ten putative I526 plants were analyzed for the RS gene using Southern blotting hybridization. The genomic DNA of nontransgenic DJ plants was used as negative control (N) (Figure 1). A signal indicating the RS gene, approximately 8.4 kb in size, was observed in all the putative I526 plants; this gene was not detected in the DJ plants (Figure 1). Accordingly, I526 plants carry the complete RS gene.

Lane P, positive control (RS cDNA); Lane N, negative control (Dongjin); Lane 1–10, Iksan526 line plants; genomic DNAs were completely digested using SacI, and the blot was hybridized using the RS cDNA probe.

### Comparison of Heading Dates and Agronomic Traits of DJ and I526 by Cultivation Year and Region

The variation in heading dates of I526 and DJ rice by cultivation year and region is presented in Table 1. The differences in the heading dates of the two varieties were 1–13 d in the Suwon region. In particular, the heading date in 2015 was later than in the previous years. The differences in the heading dates were 2–4 d during 3 y in the Iksan region and 0–9 d till 2014 in the Milyang region. Both rice varieties did not show significant differences in the heading dates. Moreover, DJ rice had earlier heading dates than I526 in all three regions.

**Table 1.** Heading dates (mean ± SD) of DJ and I526 rice in the three cultivation regions of Suwon, Iksan, and Milyang

Years	Suwon		Iksan		Milyang	
	DJ	I526	DJ	I526	DJ	I526
2013	126 ± 1.00	128 ± 2.08	112 ± 0.58	116 ± 0.58	107 ± 0.58	116 ± 1.15
2014	111 ± 0.58	112 ± 0.58	105 ± 0.58	107 ± 0.58	113 ± 0.58	113 ± 0.58
2015	115 ± 0.58	128 ± 1.00	104 ± 0.00	107 ± 1.15	108 ± 0.00	115 ± 0.00

DJ, Dongjin; I526, Iksan526.

Table 2 shows the agronomic and yield characteristics of DJ and I526 rice by cultivation year and region. No differences in agronomic traits were observed in the 3 y except in terms of panicle-related traits. Panicle length was affected by location and cultivation years and differed with the rice variety. Moreover, a difference in cultivation years substantially affected the number of panicles per hill in both varieties of rice. The highest number of panicles per hill was observed in DJ rice (13.4 panicles/hill) grown in the Milyang region in 2013, whereas the lowest number was observed in I526 rice (7.1 panicles/hill) grown in the Suwon region in 2013. However, no differences within the same region were noted. Further, there were no differences in the growth characteristics (culm length, panicle length, number of panicles/hill, and number of spikelets/panicle) in rice grown in the Iksan and Milyang regions during the study period. In addition, no difference was observed in the rough rice and brown rice yields by cultivation year (Y × V) and region (C × V).

**Table 2.** Major agronomic traits and yield characteristics of DJ and I526 rice in the three cultivation regions of Iksan, Suwon, and Milyang

Cultivation region (C)	Years (Y)	Variety (V)	Culm length (cm)	Panicle length (cm)	No. of panicles /hills	No. of spikelets /panicles	Ratio of ripened grain (%)	1,000 grain weight of brown rice (g)	Brown/ rough rice ratio (%)	Rough rice yield (kg/10a)	Brown rice yield (kg/10a)
Suwon	2013	DJ	73.2	19.0	7.8	95	91.4	25.1	81.2	558	453
		I526	74.6	20.2	7.1	107	89.3	26.2	80.6	553	446
		LSD_0.05_	ns	1.16	ns	ns	ns	0.65	0.38	ns	ns
	2014	DJ	77.0	18.3	11.1	93	85.5	23.8	80.4	591	475
		I526	78.7	20.5	10.2	96	85.1	24.3	80.0	588	470
		LSD_0.05_	ns	1.20	ns	ns	ns	ns	ns	ns	ns
	2015	DJ	82.3	19.6	10.2	99	85.3	23.6	81.6	566	462
		I526	83.5	21.1	9.0	100	84.3	24.1	80.2	583	468
		LSD_0.05_	ns	1.36	ns	ns	ns	ns	ns	ns	ns
Iksan	2013	DJ	88.9	20.8	11.2	104	93.2	23.9	82.7	606	501
		I526	87.0	20.2	11.1	111	90.0	24.6	82.3	631	520
		LSD_0.05_	ns	ns	ns	ns	ns	0.58	ns	ns	ns
	2014	DJ	84.3	20.8	11.6	87	95.3	24.0	81.9	647	529
		I526	83.3	20.9	12.0	89	92.5	24.5	82.2	656	532
		LSD_0.05_	ns	ns	ns	ns	2.05	ns	ns	ns	ns
	2015	DJ	87.7	21.0	11.3	72	93.6	25.4	80.6	631	509
		I526	87.0	22.7	9.7	89	89.1	25.3	81.5	599	488
		LSD_0.05_	ns	0.92	ns	7.72	2.00	ns	ns	ns	ns
Milyang	2013	DJ	84.8	19.7	13.4	108	92.6	22.1	82.1	687	564
		I526	84.4	20.6	11.4	112	86.6	22.4	81.5	687	560
		LSD_0.05_	ns	ns	ns	ns	5.90	ns	ns	ns	ns
	2014	DJ	84.4	21.2	10.8	89.5	92.0	21.8	80.7	616	497
		I526	72.0	19.2	13.2	93.0	90.0	24.4	81.1	593	481
		LSD_0.05_	3.46	1.02	0.77	ns	ns	0.82	ns	ns	ns
	2015	DJ	87.1	20.4	11.5	87.7	89.2	24.0	83.2	654	544
		I526	87.9	21.6	10.3	97.0	85.7	23.8	81.6	662	540
		LSD_0.05_	ns	1.02	0.76	6.86	ns	ns	1.15	ns	ns
Y × V			ns	*	*	ns	ns	ns	ns	ns	ns
C × V			ns	**	ns	ns	ns	ns	ns	ns	ns

ns, *, ** nonsignificant or significant at 0.05 and 0.01 probabilities, respectively.

DJ, Dongjin; I526, Iksan526.

### Comparison of Agronomic Traits by Cultivation Regions in Terms of Herbicide Treatment in I526

Analysis the heading dates of Basta-treated and nontreated I526 plants revealed that the heading dates of the two groups in the Iksan region were similar (0.4 d earlier in Iksan region). Further, there was no difference in the heading dates of the two groups in the other two regions (Table 3), implying that herbicide treatment did not affect heading dates. The effects of the herbicide Basta on agronomic traits in the three cultivation regions are shown in Table 4. There was no difference across all traits, except brown: rough rice ratio in the Milyang region (LSD_0.05_ = 0.84). Further, no significance differences were noted in growth and yield characteristics between the Suwon and Iksan regions. Therefore, it was confirmed that Basta-treated and nontreated I526 showed no differences in terms of growth and yield characteristics.

**Table 3.** Heading dates of I526 and 0.4% glufosinate ammonium (basta)-treated I526 (I526B) in the cultivation regions of Suwon, Iksan, and Milyang (mean ± SD)

Years	Suwon		Iksan		Milyang	
	I526	I526B	I526	I526B	I526	I526B
2015	115.3 ± 0.58	115.3 ± 0.58	107.3 ± 1.15	107.7 ± 0.58	115.0 ± 0.00	115.0 ± 0.00

I526, Iksan526.

**Table 4.** Major agronomic traits and yield characteristics of I526 and 0.4% glufosinate ammonium (Basta)-treated I526 (I526B) by cultivation regions

Cultivation region (C)	Treatment (T)	Culm length (cm)	Panicle length (cm)	No. of panicles /hills	No. of spikelets /panicle	Ratio of ripened grain (%)	1,000 grain weight of brown rice (g)	Brown/ rough rice ratio (%)	Rough rice yield (kg/10a)	Brown rice yield (kg/10a)
Suwon	I526	83.5	21.1	9.03	100.5	84.3	24.1	80.2	583	468
	I526B	81.5	20.4	8.98	103.7	84.8	23.6	79.5	562	447
	LSD_0.05_	ns	ns	ns	ns	ns	ns	ns	ns	ns
Iksan	I526	87.0	22.7	9.67	89.1	89.1	25.3	81.5	599	488
	I526B	86.0	22.0	10.67	84.2	90.8	25.1	81.6	607	495
	LSD_0.05_	ns	ns	ns	ns	ns	ns	ns	ns	ns
Milyang	I526	87.9	21.7	10.27	97.0	85.7	23.8	81.7	662	540
	I526B	88.8	21.8	9.53	97.0	87.9	23.9	82.5	646	533
	LSD_0.05_	ns	ns	ns	ns	ns	ns	0.84	ns	ns
C × T		ns	ns	ns	ns	ns	ns	*	ns	ns

ns, * nonsignificant or significant at 0.05 probabilities, respectively.

I526, Iksan526.

### Grain Yield Comparison

**Figure 2.**
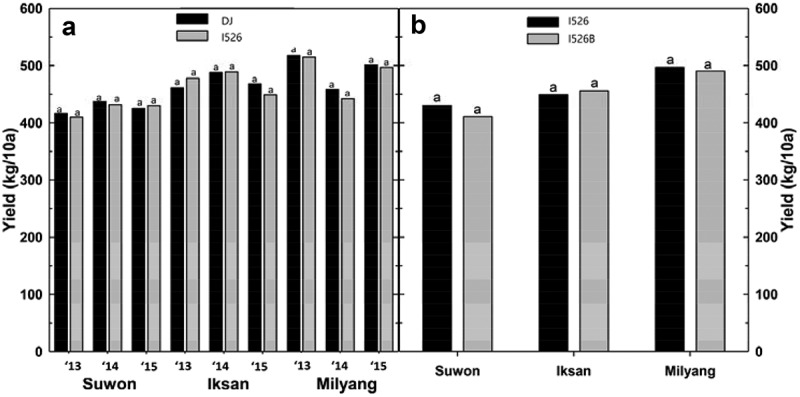
Yields of dongjin and Iksan526 rice. A: dongjin and Iksan526 rice in the three regions of cultivation during 3 y, B: Iksan526 (I526) and 0.4% glufosinate ammonium (Basta)-treated Iksan526 (I526B) in the three regions of cultivation; bars having the different letters within the same cutting time were significantly different using least significant difference 0.05

The evaluation of grain yield production of I526 and DJ rice by cultivation region and years is presented in Figure 2a. The average yields of DJ and I526 rice in the Suwon region during the 3 y were 426 and 424 kg/10a, respectively. Figure 2b shows that there was no significant difference between I526 and I526B in terms of the cultivation region. The average yields for DJ and I526 were 472 and 472 kg/10a, respectively, in Iksan and 492 kg/10a and 485 kg/10a in Milyang (Figure 2b).

### Comparison of Resveratrol Content in I526 by Cultivation Year and Region

**Figure 3.**
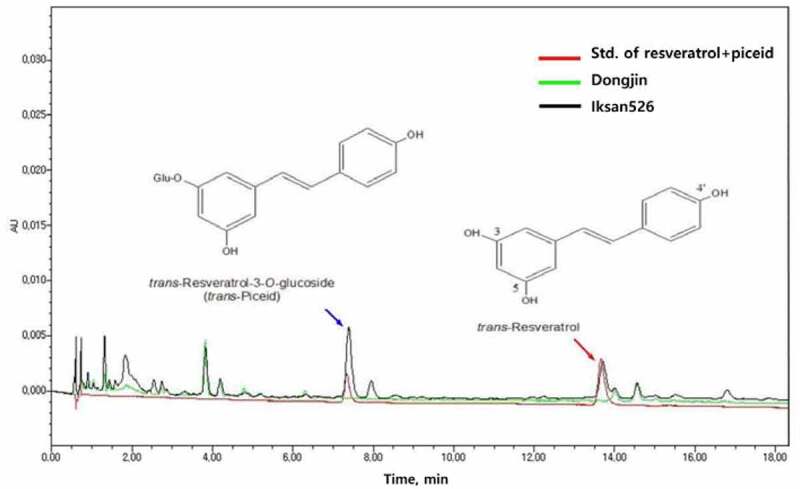
Ultra-performance liquid chromatography (UPLC) electropherograms of piceid and *trans*-resveratrol contents of Dongjin and Iksan526

**Figure 4.**
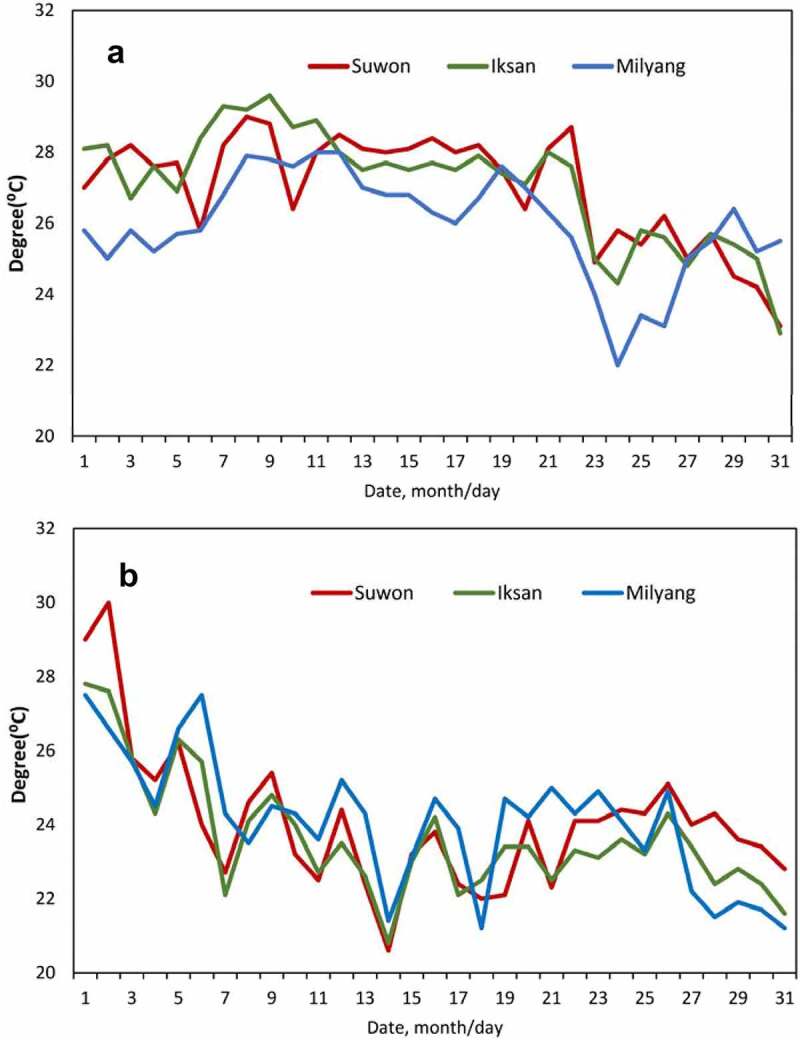
Changing temperature patterns during August 2014 (a) and 2015 (b) in the three cultivation regions of Iksan, Suwon, and Milyang

The present study confirmed that I526 produces resveratrol glycoside (piceid) and trans-resveratrol with the retention times 7.4 and 13.6 min, respectively (Figure 3). I526 from the three different regions had an average of 2.1 μg/g of trans-resveratrol. The concentration of piceid was approximately two times higher than that of resveratrol (Figure 3). The analysis of resveratrol content of I526 by cultivation region and year indicated the following results: resveratrol content increased by approximately 37% in Milyang, where the temperature rapidly decreased after heading dates around August 24 in 2014 (Figure 4a). Additionally, in 2015, resveratrol content increased by approximately 18% in Suwon, where the temperature was relatively higher than other regions during the heading time around the end of August (Figure 4b and Table 5). Total resveratrol content (μg/g) of I526 in Suwon and Milyang was 11% and 20%, respectively, higher than that in Iksan (average, 1.93 μg/g) during 2 y of observation.

**Table 5.** Content of resveratrol in iksan526 during different cultivation years in the three cultivation regions of Iksan, Suwon, and Milyang

Cultivation region	Cultivation year	Content of resveratrol (µg/g)	Index of resveratrol content
Iksan	2014	2.06 ± 0.07	100
	2015	1.80 ± 0.04	100
Suwon	2014	2.15 ± 0.08	104
	2015	2.14 ± 0.05	118
Milyang	2014	2.82 ± 0.09	137
	2015	1.81 ± 0.05	100

## Discussion

I526 was produced from the original nontransgenic DJ rice with the aim of improving rice quality by establishing a resveratrol-containing rice variety via genetic modifications. Determining the adaptability of transgenic plants is crucial to control yield stability, and it is beneficial to manage genetic resources in sustainable agriculture. To confirm the uniformity and stability of the integrated DNA in transgenic plants, Southern blotting is generally used. Using this technique, it was determined that the RS gene was present only in I526 plants, indicating that the transformation was successful in putative I526 plants.

One of important agronomic traits of rice is heading dates, which is related to the attributes of regional adaptability and yield.^18^ DJ is a mid-to-late flowering rice cultivar that is generally cultivated in the Korean peninsula (Southern plain region, 33°–44° N latitude). Based on the results, mid-to-late August, which is the summer season in Korea, is suitable for heading of both DJ and I526 rice. Heading date or flowering time in rice is an important factor for breeders to evaluate environmental adaptability in a cultivation region. Information on heading dates helps breeders in cultivation management of future crop and improve crop yield.^19,20^ Both environmental and genetic factors have been reported to influence heading dates in rice. The two major environmental factors, i.e., photoperiod and temperature, are responsible for heading time.^21^ Therefore, changing the cultivation region can result in differences in climatic zones, which can induce earlier flowering in temperate japonica rice. Genetic factors have also been reported to affect heading dates; however, their function remains unclear.^22^ Our results show that DJ rice has earlier heading dates than I526 rice in three regions; this effect is supposedly controlled by a gene in the two varieties. Genes that regulate heading dates play a key role in determining rice crop yield. The genetic mechanism underlying heading dates in rice is complicated and is controlled by a combination of photoperiod and circadian clock in addition to regulatory epigenetic mechanisms.^23,24^

Because the yield characteristics of I526 and DJ rice in multiple environments did not differ during the 3-y study period, I526 can be commercialized in the market in a manner similar to that of DJ rice. Field-testing examinations of the transgenic crop reveal its agronomic performance, thus providing an opportunity to grow the plant in a local environment.^25^ In Korea, higher production of both DJ and I526 rice was seen in the peninsula plain, including the Iksan and Milyang regions, than in with the central plain, including the Suwon region. However, the different cultivation regions did not affect the yield characteristics of I526 after treatment with Basta, indicating that I526 exhibits good geographical adaptation exhibiting better adaptation to multiple regions. However, the cultivation region and year are associated with environmental factors. Based on varieties, it is indicated that environmental factors also play a role in the expression of some agronomic traits (panicle-related attributes) in DJ and I526 rice cultivars.

The average total resveratrol content in I526 was 2.1 μg/g, which is similar to that in red wine (2.57 mg/L).^12,26^ In contrast, other sources, such as grapes, had varied total resveratrol contents ranging from 0.16 to 3.54 μg/g.^27^ Changes in resveratrol can be a consequence of glycosylation during growth and development stages. Some elicitors, such as yeast extract, upregulate glycosylation, thereby increasing the levels of piceid glycoside.^28,29^ The concentration of resveratrol varies on the basis of several factors, including weather conditions. In this study, drastic temperature changes or relatively high temperatures during the heading stage of rice resulted in increased resveratrol content in I526 rice. Among the cultivation regions in this study, I526 showed variation in resveratrol content, with the rice grown in Milyang showing the highest content. The difference in resveratrol contents of I526 among the three cultivation regions proved to be an interesting feature that must be analyzed for selecting the optimal cultivation region suitable for improving the resveratrol content of the crop in the future.

## Conclusion

Southern blotting of transgenic I526 confirmed that the RS gene was successfully transformed in the target plant. The cultivation year and region of the two rice varieties, DJ and I526, affected some agronomic traits (e.g., panicle length and number of panicles/hill), considering the interaction between different years and varieties. The herbicide Basta did not have any major effects on the agronomic traits, particularly those related to the growth and yield of I526 rice, across the three regions. Based on the data of agronomic traits during 3 y of observation in three different regions, I526 has the potential of being used as a commercialized variety in the near future due to its good yield stability and regional adaptability.
